# Proteomic and Metabolomic Characterization of SARS-CoV-2-Infected Cynomolgus Macaque at Early Stage

**DOI:** 10.3389/fimmu.2022.954121

**Published:** 2022-07-12

**Authors:** Tiecheng Wang, Faming Miao, Shengnan Lv, Liang Li, Feng Wei, Lihua Hou, Renren Sun, Wei Li, Jian Zhang, Cheng Zhang, Guang Yang, Haiyang Xiang, Keyin Meng, Zhonghai Wan, Busen Wang, Guodong Feng, Zhongpeng Zhao, Deyan Luo, Nan Li, Changchun Tu, Hui Wang, Xiaochang Xue, Yan Liu, Yuwei Gao

**Affiliations:** ^1^ Key Laboratory of Jilin Province for Zoonosis Prevention and Control, Changchun Veterinary Research Institute, Chinese Academy of Agricultural Sciences, Changchun, China; ^2^ Jiangsu Co-innovation Center for Prevention and Control of Important Animal Infectious Disease and Zoonoses, Yangzhou, China; ^3^ Department of Hepatobiliary and Pancreas Surgery, Jilin University First Hospital, Changchun, China; ^4^ Vaccine and Antibody Engineering Laboratory, Beijing Institute of Biotechnology, Beijing, China; ^5^ Key Laboratory of Organ Regeneration & Transplantation of Ministry of Education, and National-Local Joint Engineering Laboratory of Animal Models for Human Diseases, The First Hospital of Jilin University, Changchun, China; ^6^ The Key Laboratory of Pathobiology, Ministry of Education, College of Basic Medical Sciences, Jilin University, Changchun, China; ^7^ Department of Neurology, Zhongshan Hospital Fudan University, Shanghai, China; ^8^ State Key Laboratory of Pathogen and Biosecurity, Beijing Institute of Microbiology and Epidemiology, Beijing, China; ^9^ National Engineering Laboratory for Resource Development of Endangered Crude Drugs in Northwest China, The Key Laboratory of Medicinal Resources and Natural Pharmaceutical Chemistry, The Ministry of Education, College of Life Sciences, Shaanxi Normal University, Xi’an, China

**Keywords:** SARS-CoV-2 infection, immune response, cynomolgus macaque, the early stage, proteomics, metabolomics

## Abstract

Although tremendous effort has been exerted to elucidate the pathogenesis of severe COVID-19 cases, the detailed mechanism of moderate cases, which accounts for 90% of all patients, remains unclear yet, partly limited by lacking the biopsy tissues. Here, we established the COVID-19 infection model in cynomolgus macaques (CMs), monitored the clinical and pathological features, and analyzed underlying pathogenic mechanisms at early infection stage by performing proteomic and metabolomic profiling of lung tissues and sera samples from COVID-19 CMs models. Our data demonstrated that innate immune response, neutrophile and platelet activation were mainly dysregulated in COVID-19 CMs. The symptom of neutrophilia, lymphopenia and massive “cytokines storm”, main features of severe COVID-19 patients, were greatly weakened in most of the challenged CMs, which are more semblable as moderate patients. Thus, COVID-19 model in CMs is rational to understand the pathogenesis of moderate COVID-19 and may be a candidate model to assess the safety and efficacy of therapeutics and vaccines against SARS-CoV-2 infection.

## Introduction

Coronavirus disease 2019 (COVID-2019), caused by severe acute respiratory syndrome coronavirus 2 (SARS-CoV-2), has spread around the world rapidly, infected over 523 million individuals worldwide, and led to over 6.280 million deaths by June 25, 2022 (https://covid19.who.int). Unfortunately, both numbers are still increasing.

SARS-CoV-2 infection leads to COVID-19 with various severity, ranging from asymptomatic to symptomatic patients, and the latter is usually further classified as severe and moderate cases. Special attention has been given to severe COVID-19 cases who present with high fever and dry cough, pneumonia, uncontrolled inflammatory responses, and are at higher risk of death. Emerging studies have greatly unclosed the clinical and immunological characteristics, epidemiology, and pathology of severe COVID-19 patients (1, 2). For example, an epidemiological study by Ragonnet-Cronin Min et al. showed that early intervention means much lower morbidity and mortality rate of severe COVID-19 (3). Rébillard RM et al. reported that general neutropenia and lymphopenia are not specific predictors of COVID-19, while higher proportions of ALCAM^+^ monocytes, ICAM-1^+^ neutrophils, and CD38^+^ CD8^+^ T cells are often associated with higher mortality (4). Guo and colleagues performed proteomic and metabolomics profiling of sera from non-severe and severe COVID-19 patients, revealed characteristic protein and metabolite changes in severe patients (5). However, considering moderate cases account for 90% of COVID-19 patients, and timely and efficient treatment of them means better prognosis and is beneficial to alleviate the burden on medical resources, there is an urgent need to identify the underlying pathological mechanisms and effective prevention strategies for moderate cases.

Establishment of animal models is undoubtedly necessary and a great help for understanding the pathogenesis of COVID-19 (6), evaluating potential vaccines and antiviral agents against SARS-CoV-2 infection before they are used clinically (7, 8). Non-human primate models have been extensively used to investigate pathogenesis of a wide spectrum of viral diseases (9). Previous studies have reported the pathogenesis of COVID-19 in non-human primate models and compared it with MERS and SARS. The data showed that low passage clinical isolate of SARS-CoV-2 caused COVID-19-like disease in both young and aged cynomolgus macaques (CMs), with shedding virus for a prolonged period, although in the absence of overt clinical signs (10). Several groups have used rhesus macaques to simulate SARS-CoV-2 infection and pathogenesis, which usually recapitulates mild to moderate infection in humans (11, 12). These non-human primate COVID-19 models can also be effectively used to evaluate vaccines and drugs for COVID-19 therapy (12–15). Unfortunately, only rare studies performed proteomics and metabolomics analysis in non-human primate models to elucidate the mechanistic details that drive SARS-CoV-2 pathogenesis in humans (11, 16). Additionally, timely and accurate diagnosis of SARS-CoV-2 RNA makes it necessary to study the pathological characteristics of patients with very early infection (14). Thus, in this study, we established COVID-19 model in CMs and performed proteomics and metabolomics to reveal the molecular signature for COVID-19 pathogenesis at very early stages of SARS-CoV-2 infection (7 days post infection).

## Materials and Methods

### Animals and Experimental Procedures

The CMs used in this study were bred and provided by the Laboratory Animal Center, Academy of Military Medical Sciences (Beijing). All animals were confirmed to be specific pathogens (especially SARS-CoV-2) free before the experiment. To establish the model of COVID-19, CMs were anesthetized with Zoletil 50 and inoculated with a dose of 4.7 × 10^6^ TCID_50_/mL SARS-CoV-2 *via* a combined intranasal (0.25 mL per nasal), intratracheal (4.0 mL), and ocular conjunctival routes (0.1 mL per eye). The control CMs were inoculated with an equivalent dose of DMEM. On 2, 4 and 6 days after infection, the nasal, throat, and anal swabs were collected and incubated in 1 mL of PBS containing 1000 μg/mL streptomycin and 1000 U/mL penicillin. The body weight and temperature were measured every other day. The UCT-2112 temperature probes (American Health & Medical Supply International Corp., USA), which were injected interscapularly into the CMs before the experiment, were used to monitor the body temperature. On day 7 post infection, all animals were euthanized and tissues including nasal turbinate, trachea, different lobes of lung, heart, spleen, kidney, colon, brain, liver, and testes were collected to detect the viral loads. All the experiments were done in Animal Biosafety Level 3 (ABSL3) at the Key Laboratory of Jilin Province for Zoonosis Prevention and Control, Institute of Military Veterinary Medicine, according to the protocols approved by the Administrative Committee on Animal Welfare of the Institute of Military Veterinary.

### RNA Extraction and qRT-PCR

Total RNA was isolated from 200 μL of samples by using Magnetic Viral DNA/RNA Kit (Tiangen Biotech, Beijing, China). After synthesizing cDNA with reverse transcriptase (Invitrogen, Carlsbad, CA, USA), qPCR was conducted by Bio-Rad CFX96 Real-time PCR system (Bio-Rad, Hercules, CA) following cycling protocol: 50°C for 20 min, followed by 95°C for 3 min and then by 45 cycles of 95°C for 5 s, and 57°C for 45 s. The ORF1ab gene-specific primers (forward, 5’-CCCTGTGGGTTT TACACTTAA-3’; reverse, 5’-ACGATTGTGCATCAGCTGA-3’) and probe 5’-FAM-CCGTCTGC GGTATGTGGAAAGGTTATGG-BHQ1-3’. The N gene-specific primers (forward, 5’-GGGGAAC TTCTCCTGCTAGAAT-3’; reverse, 5’-CAGACATTTTGCTCTCAAGCTG-3’) and probe 5’-FAM-TTGCTGCTGCTTGACAGATT-TAMRA-3’ were used according to the information provided by the National Institute for Viral Disease Control and Prevention, China. The gene-specific primers (sgLead-forward: 5’-CGATCTCTTGTAGATCTGTTCTC-3’; reverse: 5’-ATATTGCAGCAGTAC GCACACA-3’) and probe 5’-FAM-ACACTAGCCATCCTTACTGCGCTTCG-BHQ1-3’ were used for E gene subgenomic mRNA quantitation.

### Luminex Assay of Inflammatory Cytokines and Chemokines in Macaque Serum

Peripheral blood samples and sera were collected from all Macaques, the levels of 30 cytokines and chemokines like interleukin (IL)-2, IL-6, IL-8, IL-10, IL12, IL-17, IL-23, MCP-1, IP-10 (CXCL10), MIG, MIP-1β (CCL4), CD40, and SDF-1α etc. were determined by Luminex multi-factor detection platform (eBioscience ProcartaPlex, Thermo Fisher Scientific, Waltham, MA, USA) according to the manufacturer’s protocol.

### Flow Cytometry and Blood Routine Analysis

Samples of EDTA anticoagulated peripheral blood (6 mL) were collected from control or SARS-CoV-2–infected CMs. Then, diverse cell populations were measured by flow cytometry according to the manufacturer’s instructions with the following monoclonal antibodies: anti-CD3-PE-Cy7 (BD Biosciences, 557917), anti-CD4-APC (Biolegend, 317415), anti-CD8-PE (Biolegend, 301008), anti-CD20-PE (Biolegend, 302306), anti-CD16-PE (Biolegend, 302007), and anti-CD56-APC (Biolegend, 318309). Samples were analyzed on a BD FACS Canto II flow cytometry system (BD Biosciences). For blood routine analysis, anticoagulant venous blood collected at indicated time points were subjected to five classification hematologic analyzer (Mindray, China).

### Histological Evaluation

For histopathologic examination, tissue samples including lung, trachea, salivary gland, heart, liver, kidney, and brain, were 4%-paraformaldehyde-fixed, paraffin-embedded, sectioned at 5 μm, and subjected to hematoxylin and eosin (H&E) and immunohistochemical (IHC) staining. For IHC staining, the primary antibodies used in the experiments including anti-CD4, CD8, CD14, CD16, CD20, CD64, and Myeloperoxidase (MPO) were all purchased from Abcam. Images of H&E and IHC stained slides were captured with an Olympus BX43 microscope (Olympus).

### Proteome Analysis

A 4D-Label free quantitative proteomics analysis was performed using tandem MS/MS in Q Exactive™ Plus coupled online to an EASY-nLC 1000 UPLC system (Thermo) in JingJie Sciences Company (Hangzhou, China). Total proteins were extracted from the frozen lung samples by adding 4 times the volume of 10% TCA/acetone at -20°C and precipitating for more than 4 h. After centrifugation at 4500 g for 5 min, the precipitate was collected and washed 2-3 times with pre-cooled acetone. Then, the pellet from each specimen was reconstituted with lysis buffer (8 M urea, 3 μM TSA, 50 mM NAM, 1% protease inhibitor), and the protein concentration was determined by BCA kit. Equal amount of each protein sample was then enzymatically lysed and adjusted the volume to the same with the lysate. Then, samples were reduced by 5 mM dithiothreitol (DTT) at 56°C for 30 min and alkylated by 11 mM iodoacetamide (IAA) at room temperature for 15 min in the dark, respectively. The alkylated sample was then transferred to an ultrafiltration tube and centrifuged at 12000 g at room temperature for 20 min, followed by replacement with 8 M urea and replacement buffer for 3 times. After that, protein samples were digested by incubation with trypsin at a ratio of 1:50 (protease: protein, m/m) for overnight. Finally, the fragmented peptides were recovered by centrifugation at 12000 g for 10 min at room temperature, and the peptides were recovered with ultrapure water once, and the two peptide solutions were combined. For LC-MS/MS analysis, the tryptic peptides were dissolved in solvent A (0.1% formic acid (FA), 2% acetonitrile in water) and loaded onto a home-made reversed-phase analytical column (25-cm length, 100 μm i.d.). The peptides were separated by eluting the column with a gradient from 6% to 24% solvent B (0.1% FA in 98% acetonitrile) over 70 min, 24% to 35% in 14 min and reached up to 80% in 3 min, then keeping at 80% for 3 min, all at a constant flow rate of 450 nL/min on a nanoElute UHPLC system (Bruker Daltonics). The peptides were then subjected to capillary source followed by the timsTOF Pro MS (Bruker Daltonics). The timsTOF Pro was operated in a parallel accumulation serial fragmentation (PASEF) mode with the electrospray voltage 2.0 kV. Precursors and fragments were analyzed at the TOF detector, and the MS/MS scan ranged from 100 to 1700 m/z. Precursors with charge states 0 to 5 were selected for fragmentation, and 10 PASEF-MS/MS scans were acquired per cycle. The dynamic exclusion was set to 30 s. For database search, The MS/MS data were processed by MaxQuant search engine (v.1.6.6.0). Tandem mass spectra were searched against the *Macaca_mulatta*_9544 database (45179 entries) concatenated with reverse decoy database. Trypsin/P was specified as cleavage enzyme allowing up to 2 missing cleavages. The mass error tolerance for precursor ions was set to 20 ppm in both First search and Main search, and fragment ions mass error tolerance was also set to 20 ppm. Carbamidomethyl on Cys was specified as fixed modification, and acetylation on protein N-terminal and oxidation on Met were specified as variable modifications. The false discovery rate (FDR) (strict) was adjusted to < 1%.

### Metabolome Analysis

Plasma/serum samples were thawed on ice, and 3 volumes of ice-cold methanol was added. Samples were agitated for 3 min and centrifuged at 12,000 rpm and 4°C for 10 min. Then the supernatant was collected and centrifuged at 12,000 rpm and 4°C for 5 min. The final supernatant was collected for LC-MS/MS analysis using an LC-ESI-MS/MS system (UPLC, Shim-pack UFLC SHIMADZU CBM A system, https://www.shimadzu.com/; MS, QTRAP^®^ System, https://sciex.com/) in JingJie PTM BioLab Co. Ltd. (Hangzhou, China). The analytical conditions were as follows, UPLC: column, Waters ACQUITY UPLC HSS T3 C18 (1.8 µm, 2.1 mm × 100 mm); column temperature, 40°C; flow rate, 0.4 mL/min; injection volume, 2 μL; solvent system, water (0.1% FA): acetonitrile (0.1% FA); gradient program, 95:5 V/V at 0 min, 10:90 V/V at 11.0 min, 10:90 V/V at 12.0 min, 95:5 V/V at 12.1 min, 95:5 V/V at 14.0 min. Finally, samples were analyzed with ESI-QTRAP-MS/MS. LIT and triple quadrupole (QQQ) scans were acquired on a triple quadrupole-linear ion trap mass spectrometer (QTRAP), QTRAP^®^ LC-MS/MS System, equipped with an ESI Turbo Ion-Spray interface, operating in positive and negative ion mode, and controlled by Analyst 1.6.3 software (Sciex). The ESI source operation parameters were set as follows: source temperature 500°C; ion spray voltage 5500 V (positive), -4500 V (negative); ion source gas I, gas II, and curtain gas were set at 55, 60, and 25 psi, respectively; the collision gas was high. Instrument tuning and mass calibration were carried out with 10 and 100 μM polypropylene glycol solutions in QQQ and LIT modes. A specific set of MRM transitions was monitored for each period according to the metabolites eluted within this period. For combined omics analysis, we extracted the proteins and metabolites with differential expression and performed pair-wise correlation network analysis to identify co-regulated nodes. Each node in blue circle represented a protein and the box nodes of different colors represented different kinds of metabolites. We calculated the Spearman’s correlation coefficient(ρ) between each protein and metabolite based on their abundance levels and then we showed the nodes between which the absolute value of ρ is greater than 0.6.

### Statistical Analysis

All data were analyzed with GraphPad Prism 8.0 software. For any test, a *P* value of < 0.05 was considered to be significant. Statistical significance is shown as *P < 0.05, and **P < 0.01 compared between indicated groups.

## Results

### Establishment and Identification of COVID-19 Model in CMs

**Figure 1:**
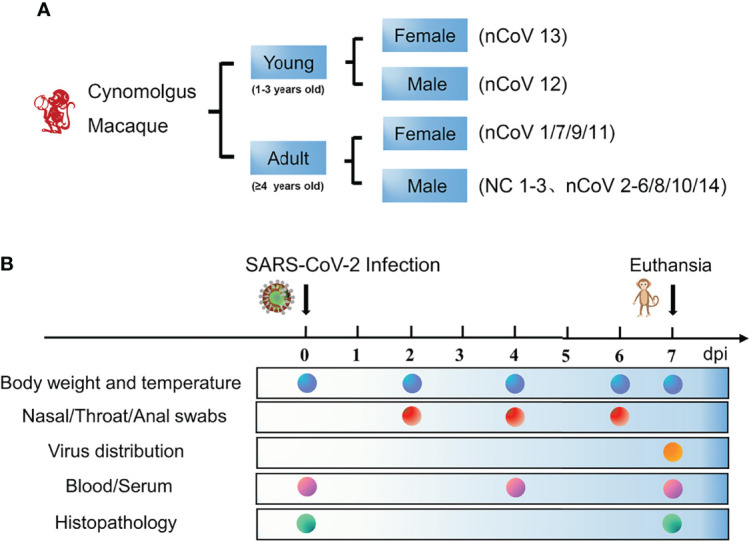
Schematic of the study design and clinical signs of SARS-CoV-2 infection in CMs. **(A)** Two age groups of monkeys (14 in total) were selected for this study to assess their ability to establish COVID-19 model. The monkeys were randomly assigned into two groups, the detail of age and sex were demonstrated. **(B)** The body weight and temperature, swabs, tissues and blood were collected at the indicated time points for evaluation of clinical symptoms, viral shedding and replication, and host responses to SARS-CoV-2. CMs, cynomolgus macaque; NC, negative control; nCoV, SARS-CoV-2.

**Figure 2:**
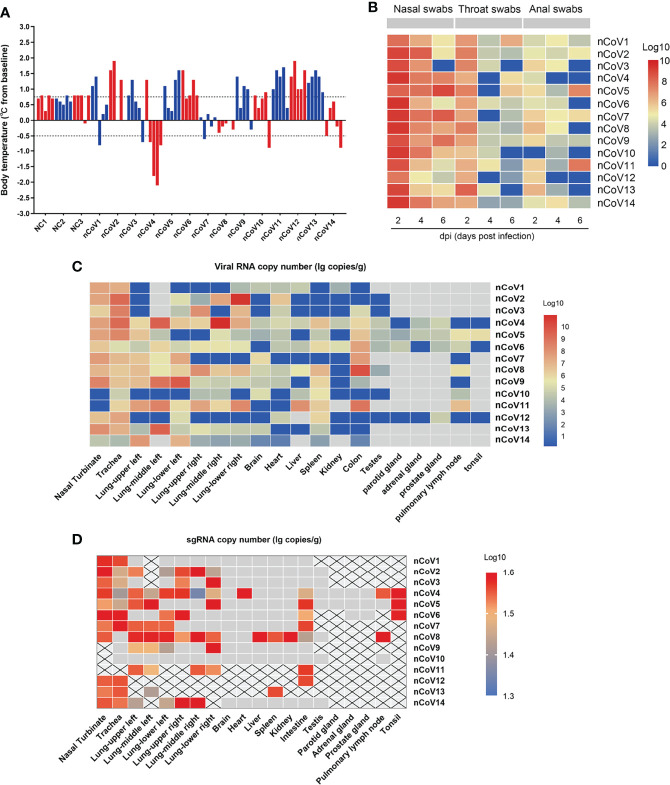
Body temperature, viral shedding and replication in CMs inoculated with SARS-CoV-2. **(A)** The body temperature of each monkey was monitored and recorded at above indicated time points after SARS-CoV-2 inoculation (0, 2, 4, 6, 7dpi). The body temperature changes were calculated by subtracting the baseline (37°C) from each. **(B)** Every two days after virus inoculation, swabs (nasal, throat, and anal) were collected from the monkeys for quantification of virus genomic RNA *via* qRT-PCR. **(C)** Tissue samples were harvested from necropsied animals at 7 dpi for detection of viral load by qRT-PCR. The viral copies were indicated as a log_10_ value, and the heatmap was prepared *via* heatmap illustrator in TB tools. **(D)** E gene subgenomic viral RNA transcripts were examined by qRT-PCR. qRT-PCR, real-time quantitative-polymerase chain reaction; sgRNA, subgenomic viral RNA.

We inoculated 12 adult CMs (11–17 years old, mean = 14 years) and 2 young CMs (1–3 years old, mean = 2 years) with a dose of 4.7 × 10^6^ TCID_50_/mL SARS-CoV-2 (BetaCoV/Beijing/IME-BJ05/2020) (17), administered by intranasal (IN, 0.25 mL per nasal), intratracheal (IT, 4.0 mL), and conjunctival (CJ, 0.1 mL per eye) routes (18, 19). The detailed information of infection and detection time was demonstrated in **Figures 1A, B**. The body temperature was monitored every other day after SARS-CoV-2 challenge from 0 to 7 days. As shown in **Figure 2A**, an increased body temperature of 0.75°C was observed in 10 out of 14, whereas a decrease of 0.5°C was observed in 3 out of 14 SARS-CoV-2-infected CMs. To determine the infection kinetics, viral load and shedding in swabs and tissues were detected by qRT-PCR at indicated time points. We found that the levels of viral genomic RNA in nasal swab samples from all CMs reached peak at 2 dpi (median: 5.4 × 10^11^ copies/μL), then 8 of 14 decreased and 6 remained high levels. The peak values were observed in throat swabs at 2 dpi, the values were lower than those in nasal swabs, which decreased to 2.45 × 10^6^ copies/μL (median value) at 6 dpi. Meanwhile, the viral RNA levels in anal swab samples were lower than that in nasal and throat swabs (**Figure 2B**).

Moreover, we evaluated the SARS-CoV-2 viral RNA copies in various tissues of the infected CMs at 7 days after infection. As shown in **Figure 2C**, viral RNA could be detected in 15 of 20 tissues with highest level found in the nasal turbinate, trachea and lung, but much lower viral RNA copies were measured in brain, heart, liver, spleen, kidney, testes, parotid gland, adrenal gland, prostate gland, pulmonary lymph node and tonsil. Moreover, a relatively high level of viral copies could be detected in colon in 7/14 infected CMs.

To verify the active virus replication, the viral E gene subgenomic mRNA were further examined (20). As indicated in **Figure 2D**, except kidney, the viral could replicate in several tissues including nasal turbinate, trachea, lung, spleen, colon, prostate, pulmonary lymph node and tonsil, although with the limited replication efficacy. These results confirmed that SARS-CoV-2 could replicate in multiple tissues of CMs besides the respiratory route, the fecal–oral route may be involved in viral transmission (21, 22), which supported that establishment of COVID-19 in CMs is an ideal model for SARS-CoV-2–associated study.

### Clinical Features of COVID-19 in CMs

**Figure 3:**
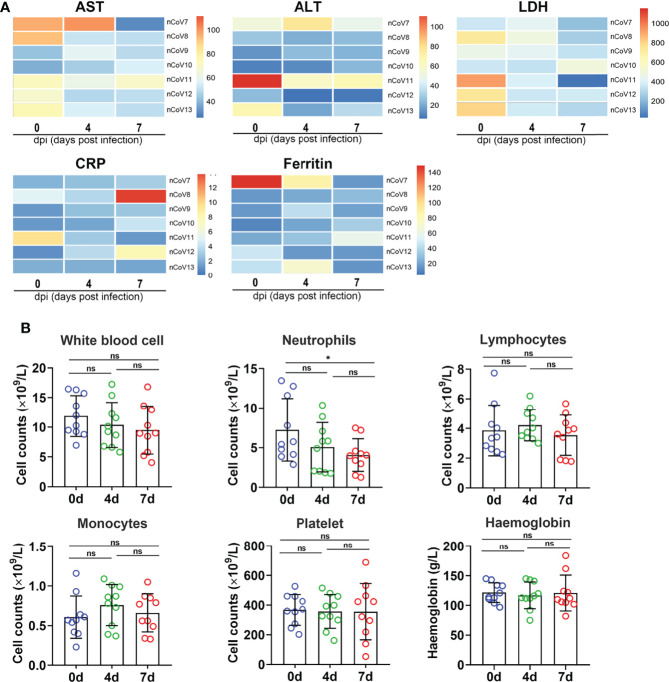
Clinical features in monkeys inoculated with SARS-CoV-2. **(A)** The infection indicators (AST, ALT, LDH, CRP and Ferritin) were examined at indicated time points and compared with each other. The 0 dpi (before infection) of each monkey was put as the control of itself. The heatmap was prepared *via* heatmap illustrator in TB tools. **(B)** The counts and proportion of indicator cells in PBMC were detected at indicated time points in macaques before and after SARS-CoV-2 challenge, and the analysis was plotted in GraphPad Prism 8.0.1. *p<0.05. ns, not significant. AST, aspartate transaminase; ALT, alanine transaminase; LDH, lactate dehydrogenase; CRP, C-reactive protein; nCoV, SARS-CoV-2; PBMC, peripheral blood mononuclear cell.

Several infection markers like alanine aminotransferase (ALT), aminotransferase (AST), lactate dehydrogenase (LDH), high-sensitivity C-reactive protein (CRP), and ferritin were measured to evaluate the severity of COVID-19 in CMs. Results demonstrated that ALT, CRP, and ferritin values were distributed in normal range in almost all the infected CMs, which are similar as those observed in moderate COVID-19 patients (23, 24), but are apparently different from severe ones, in which all these indicators increased significantly (5). As to AST and LDH, significant decreases were found in about 57.1% (4/7) of infected CMs at 7 dpi when compared with control CMs. (**Figure 3A**).

We then isolated peripheral blood mononuclear cell (PBMC) from CMs blood and analyzed the changes of white blood cell count (WBC), lymphocyte count (LYC), monocyte count, absolute neutrophils count (ANC), platelet count (PLT), and hemoglobin (HGB). As shown in **Figure 3B**, WBC decreased slightly from 11.11 to 8.94 × 10^9^/L upon SARS-CoV-2 infection. LYC remains unchanged (about 3.74 × 10^9^/L) in infected CMs at 7 dpi, which was consistent with the evidence that lymphopenia (LYC < 0.8 × 10^9^/L) was always developed in severe (72.7%) but not in moderate COVID-19 cases (25). ANC were found to be decreased in challenged CMs (4.02 × 10^9^/L) when compared with the healthy controls (5.58 × 10^9^/L, p<0.05), as reported in moderate COVID-19 patients (2.6-4.4 × 10^9^/L) (26). Additionally, monocytes increased slightly, while HGB and PLT appeared almost unchanged in infected CMs.These data collectively suggested that PBMC indicators are more semblable in CMs models at early stage as that in moderate patients, the obvious turbulence of hematological indicators such as lymphopenia and thrombocytopenia that appeared in very severe and severe COVID-19 patients, is obviously weakened in infected CMs.

### Immunological Features of COVID-19 Model in CMs

**Figure 4:**
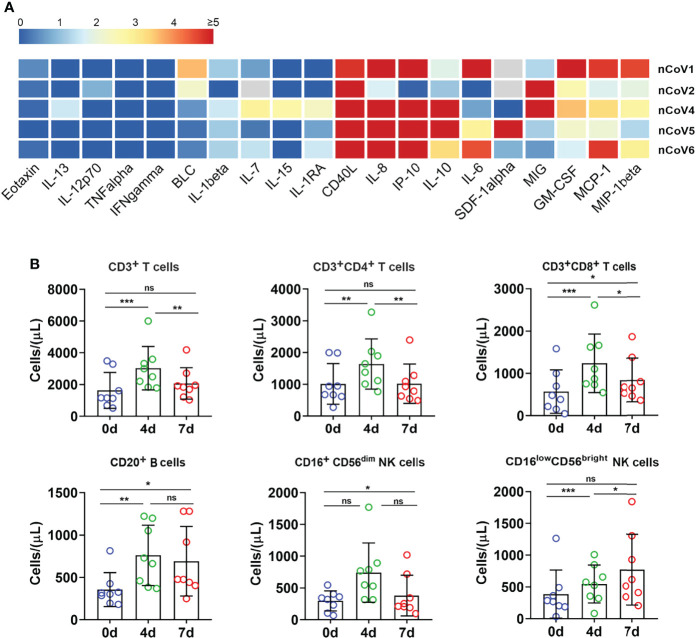
Inflammatory cytokines and immune cell subpopulations in COVID-19 macaques. **(A)** Inflammatory cytokines in serum samples from macaques before and after challenge were measured by Luminex multiplex assays as described in the “Materials and methods” section. The scale bar indicates the change-fold of the cytokines at 7dpi, compared with themselves at before exposure. **(B)** The proportion of immune cells including CD3^+^, CD4^+^, and CD8^+^ T lymphocytes, CD20^+^ B lymphocytes, and NK cells were analyzed by flow cytometry. *p<0.05; **p<0.01; ***p<0.005. ns, not significant.

To gain insight into the immunological features of COVID-19 in CMs, we applied a Luminex multi-factor detection to assess the immune cytokines alteration in serum samples of CMs before and post SARS-CoV-2 inoculation. As showed in **Figure 4A**, sCD40L, IL-8, GM-CSF, IP-10, MCP-1, and MIG and IL-10 significantly increased (p<0.01), while IL-6, IL-1β, IL-7 and MIP-1β moderately increased in infected CMs (p<0.05). Notably, IL-10, the traditional Th2 cytokine which usually exerts dual effects on T cells in terms of inhibiting Th1 cell production of IL-2, IFNs as well as TNF-α and enhancing the proliferation and cytolysis activity of NK and CD8^+^ T cells, elevated in infected CMs, which indicated the susceptibility to COVID-19. Emerging studies also reported that IL-10 is a well-known marker of COVID-19 severity in the clinical setting (27, 28). Several cytokines like IL-12, IL-13, IFN-γ, and IL-1RA, which elevated significantly and predicted severe cases, were unchanged or weakly increase in our CMs model. Therefore, these cytokines may coordinately induce the anti-virus and inflammatory response, explaining why SARS-CoV-2 infection induces moderate symptoms and thoracic injury in CMs.

Then, immune cells populations in PBMC of CMs were investigated by flow cytometry. As showed in **Figure 4B**, the absolute numbers of total T lymphocytes, CD4^+^ T and CD8^+^ T cells significantly increased at 4 dpi. Then, CD4^+^ T cells decreased to basal level, while CD3^+^ and CD8^+^ T cells hold a slightly higher level than the basal value at 7 dpi. Similar trends were found for CD20^+^ B lymphocytes. Analysis of NK cells showed an increase of secretory population (CD16^low^CD56^bright^) upon infection, then decreased to basal level at 7 dpi, while cytotoxic CD16^+^CD56^dim^ NK cells remained relatively stable after infection as compared to before infection. These data indicated that SARS-CoV-2 infection stimulated T, B and NK cells proliferation and activation in CMs at very early stage, followed by quickly decrease to the basal level. In contrast, the absolute counts of total T lymphocytes, CD4^+^, CD8^+^ T subpopulations and NK cells were all reduced potently in the vast majority of severe COVID-19 patients, even below the lower limit of normal, and these reductions are closely-associated with the severity of COVID-19 cases (29). In addition, the proportion of B cells was significantly higher in severe cases (20.2%) than in moderate ones (10.8%) (30). Thus, these data indicated that SARS-CoV-2 triggered rapid, timely, moderate and controllable immunological response in CMs, and hence avoided excessive immune responses appeared in severe COVID-19 patients.

### Histopathological Change and Immune Cells Infiltration

**Figure 5:**
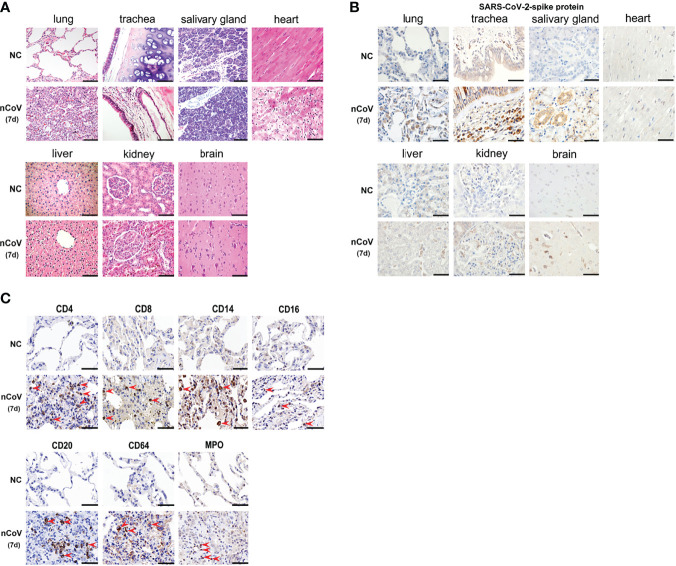
Pathological evaluation, viral distribution and host response in tissues of COVID-19 CMs. **(A)** After necropsy, different tissue samples were cut and fixed in 10% neutral buffered formalin for H&E staining, followed by microscopic inspection. **(B)** SARS-CoV-2–Spike antibody was used to evaluate the viral load and distribution in different tissues. **(C)** Infiltration of immune cells was examined in the lung tissues. The red arrowheads indicate corresponding positive cells in the pulmonary airspace. Scale bar=100 μm. NC, negative control; nCoV, SARS-CoV-2; 7d, 7 days post infection; CMs, cynomolgus macaque.

Noting the results of serum cytokines and chemokines, H&E and immunohistochemical (IHC) staining were performed to confirm the inflammatory response and injury in lung tissues of infected CMs. The moderate histopathological characteristic for diffuse alveolar damage were observed in lung tissues, which represented as interstitial pneumonia, widened alveolar septum, hyperemia of alveolar wall capillaries, mainly infiltration of macrophages, neutrophils, accompanied with scattered eosinophils, proliferation of fibroblast-like cells and deposition of powdered matrix (**Figure 5A**). Focal consolidation was frequently observed in some areas, with coagulation necrosis occasionally, and inflammatory exudation in alveolar cavity. Meanwhile, monocytes dominated inflammatory infiltration were observed in heart, and renal interstitium, no significant histopathological changes were observed in other organs, such as salivary gland, liver, brain, tonsil, adrenal gland, prostate, spleen, intestine and testicles (**Figure 5A**).

The SARS-CoV-2-Spike protein was then examined in diverse tissues of CMs. Strong positive staining was predominantly observed in alveolar epithelial cells, vascular endothelial cell, alveolar macrophages, salivary gland, hepatic Kupffer cells, and part of glomerulus cells. Weak positive staining was observed in brain and kidney, and no staining was observed in heart (**Figure 5B**). These data were consistent with the results of viral genomic RNA copies in **Figure 2C**.

IHC was further employed to detect infiltration of immune cells in lung tissues of CMs. As showed in **Figure 5C**, moderate infiltration of CD4^+^ T, CD8^+^ T cells, CD14^+^ monocytes, CD64^+^ and MPO^+^ neutrophils, CD16^+^ NK cells, and CD169^+^ macrophages were detected in CMs at 7 dpi. Meanwhile, no significant increase of CD20^+^ B cells were observed in challenged CMs. Most of these are consistent with the distribution of immune cells in PBMC, except neutrophil, which increased apparently in challenged lung tissues (p<0.05), whereas decreased in PBMC. These data supported that adequate innate and adaptive immunity play critical anti-viral roles, and recruitment of circulating neutrophils to the infection site is a main characteristic of COVID-19 models in CMs.

### Proteomic and Metabolomic Alterations Post SARS-CoV-2 Exposure

Although accumulating proteomics and metabolomics analysis have been performed in COVID-19 patients (5), rare studies paid attention to omics changes at the initial stage of viral infection, especially for moderate cases. Considering the viral detection is becoming more efficient, and timely and efficient treatment of moderate cases (which account for 90% of COVID-19 patients) means better prognosis and alleviation of burden on medical resources, we thus tried to explore the pathogenesis of COVID-19 in CMs models at early stage of infection. We used 4D Label-free proteomics and ultra-performance liquid chromatography/tandem mass spectrometry (UPLC-MS/MS) targeted metabolomics approaches to analyze the lung tissues and sera samples, respectively. Altogether, 4493 proteins and 500 metabolites were identified and quantified.

**Figure 6:**
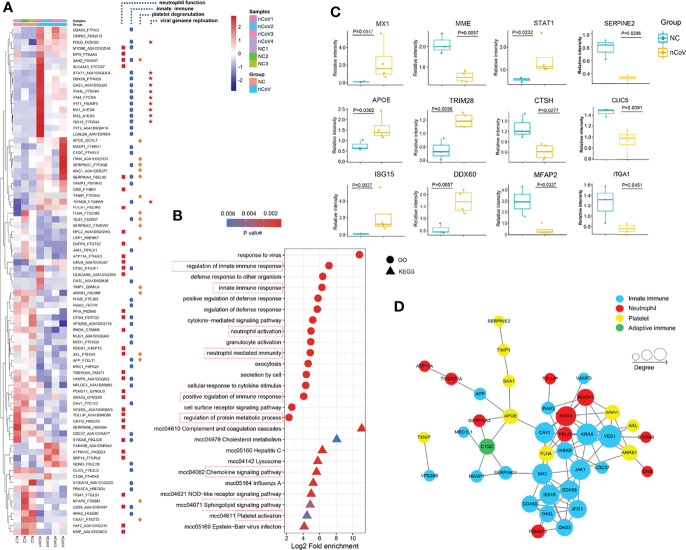
Dysregulated Proteins in lung tissue of monkeys inoculated with SARS-CoV-2, and statistics of functional enrichment. **(A)** Heatmap of 83 selected proteins whose regulation concentrated on four enriched pathways. **(B)** The median CV values was analyzed to confirm the proteomics data with good degree of consistency and reproducibility (median<0.25). **(C)** The expression level change of nine selected proteins with significant difference before and after inoculated with SARS-CoV-2. **(D)** The histogram of GO terms enriched in biological process, cellular component and molecular function. CV, coefficient of variance; GO, Gene Ontology.

We found that 194 proteins were differentially expressed in lung tissue of nCoV2 CMs (65 increased *vs* 129 decreased, >1.5-fold, **Figure 6A**) as compared with NC CMs. Target and functional analyses showed that 83 of these proteins belong to four major pathways, namely innate immune response (44 proteins), neutrophil activation and degranulation (33 proteins), platelet degranulation (17 proteins), and viral genome replication (11 proteins). Consistently, the Gene ontology (GO) and Kyoto Encyclopedia of Genes and Genomes (KEGG) analysis data also revealed that innate immune response, neutrophil and platelet activation pathways are involved in moderate COVID-19 onset in CMs (**Figure 6B**). For all differentially expressed proteins, 9 most significantly changed innate immune-associated proteins were shown in **Figure 6C**. most of the differentially expressed genes (DEGs) in these four signal pathways can form a protein-protein interaction (PPI) network, so as to potently and jointly regulate the process of COVID-19 (**Figure 6D**). In addition, GO analysis was performed to produce a further protein classification and assess gene enrichment sets, in biological process, cellular component, and molecular function.

**Figure 7:**
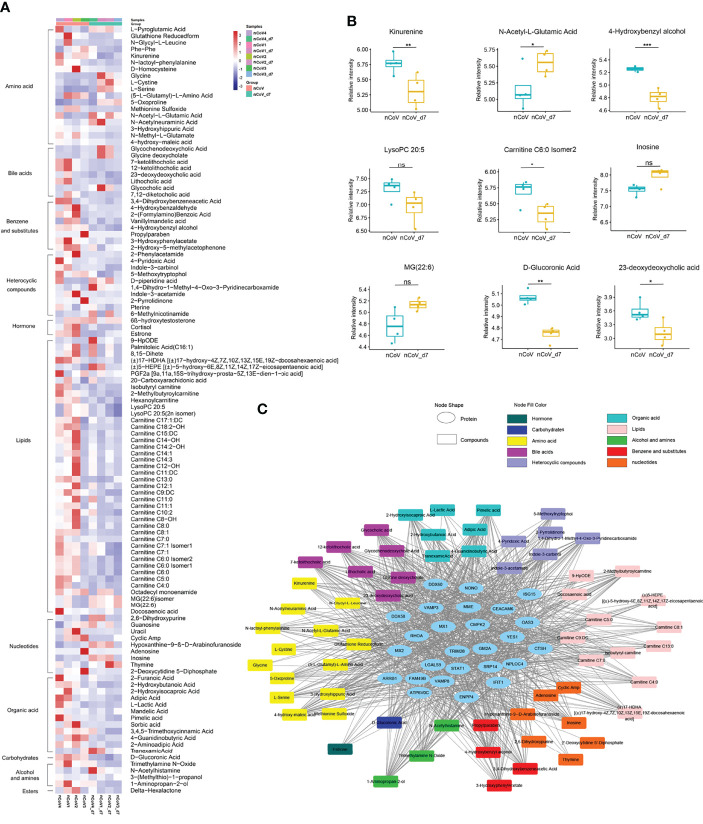
Dysregulated metabolites in sera of CMs inoculated with SARS-CoV-2, and statistics of functional and pathway enrichment. **(A)** Heatmap of 116 most significantly regulated metabolites belonging to 11 major classes as indicated. **(B)** The expression level change of nine selected regulated metabolites with significant difference before and after inoculated with SARS-CoV-2. **(C)** Scatter plot of KEGG enrichment analysis between nCoV *vs.* nCoV-d7 with significant changes. CMs, cynomolgus macaque; nCoV, SARS-CoV-2; nCoV_7d, 7 days post SARS-CoV-2 infection; KEGG, Kyoto encyclopedia of genes and genomes. *p<0.05; **p<0.01; ***p<0.005. ns, not significant.

Meanwhile, 131 metabolites were found to be significantly changed in nCoV2 CMs (**Figures 7A-C**). Among them, 116 metabolites were involved in the four biological processes revealed in the proteomic analysis, and we summarized both proteomics and metabolomics data in the following sections and indicated their function in COVID-19 onset.

### SARS-CoV-2 Infection Leads to Neutrophil Recruitment and Activation

As the most abundant white blood cell in humans, neutrophils play critical roles in antiviral defense of innate immune system. But through degranulation and formation of neutrophil extracellular traps (NETs), they can be cytotoxic during severe pneumonia (31). Constantly updated data proved that a fulminant neutrophilia is often observed in severe COVID-19 cases, which could be a symbol of excessive inflammatory response and associated with unfavorable prognosis (26, 32, 33), whereas neutropenia was more common in non-severe group (34, 35), which is consistent with our CM models. Hence, it is urgent to unravel the roles of neutrophils in the moderate cases of COVID-19.

We obtained 33 altered proteins (12 upregulated, 21 downregulated) that are involved in neutrophil recruitment, activation, and degranulation by target-function analysis (**Figure 6A**) in the current model. ATP6V0C, a modulator of neutrophil degranulation and activation, were increased 6.16- and 10.61-fold, respectively. Whereas AXL, which is important for neutrophil infiltration and COVID-19 therapy *via* regulating TGF-β and PI3K signaling pathways (36, 37), also works as a candidate receptor for SARS-CoV-2 entry (38), was significantly reduced about 3-fold in infected CMs as compared with the healthy control. Glia maturation factor-γ (GMFG), decreased by 0.45-fold, could regulate directional migration of neutrophils through regulation of actin cytoskeletal reorganization and integrin-mediated adhesion (39). As known, NETosis is a critical pathologic driver of direct lung injury in COVID-19 patients (40, 41). Of these 33 proteins, LGALS9, MPO, S100A8, C1QC, C1QB, and CTSC were NETs-associated, and their changes were not as obvious as in severe COVID-19 patients (42).

Considering the facts that neutrophil-attracting chemokines like IL-8, MCP-1, and MIP-1β are dramatically upregulated (**Figure 4A**); dozens of neutrophil recruitment, activation, and degranulation-associated genes were significantly changed (**Figure 6A**); neutrophil counts were reduced in PBMC of infected-CMs, these data collectively indicated that the migration and adhesion capacity of neutrophils to lung tissues were tightly- and timely-regulated in infected CMs.

### Innate Immune Response Was Potently Activated by SARS-CoV-2 Infection

Activation of innate immunity may assist rapid recognition, suppressed viral evasion and short-term retention of SARS-CoV-2. We identified 44 proteins from all 194 proteins significantly altered in infected-CMs which are known to be responsive to innate immunity. A few representative examples were showed in **Figures 6A, C**. Most of these proteins are encoded by interferon-stimulated genes (ISGs), therefore, our data strongly supported that interferon (IFN) signaling pathway-associated anti-viral action play an essential role in the model. STAT1, a crucial modulator of IFN signaling, can’t be avoided in antiviral innate immunity. A study showed that hACE2 transduced STAT1^-/-^ C57BL/6 mice exhibited enhanced inflammatory cell infiltration, and delayed SARS-CoV-2 clearance (43). In our model, STAT1 was significantly upregulated 2.62-fold as compared with the NC CMs. ISG15 was upregulated up to 20.51-fold. MX1 and MX2, downstream targets of ISG15 which play important anti-viral effects on a variety of RNA and DNA viruses, were upregulated 21.44- and 56.85-fold, respectively (44). As a family of IFN-induced antiviral proteins, IFITs potently restrict viral infection by recognizing virus-derived exogenous RNAs (45, 46). IFIT1 and IFIT2 were upregulated 7.8- and 5.7-fold, respectively. Thus, it is rationale to predict that in moderate cases, SARS-CoV-2 induced ISGs activation, the latter one leaded to the constitutively expression of MX1/2 and IFIT1/2, which then extended and enhanced the anti-viral activity of innate response, thereby alleviate the symptoms of COVID-19 and overcome SARS-CoV-2 viral evasion in CMs.

DDX58 (also known as RIG-I) can initiate a signaling cascade to start an inflammatory and type-1 IFN responses once recognizing pathogen-associated molecular patterns (PAMP), like virus-derived dsRNA. DDX58 was upregulated (3.6-fold) in infected CMs. DDX60, another member of RIG-I signaling pathway, is essential for viral RNA degradation (47), was also remarkably upregulated (3.1-fold, **Figure 6C**). TRIM28, which is involved in innate immunity, has been identified recently to be a regulator of ACE2 expression and SARS-CoV-2 entry (48), also increased significantly in our model.

Although lots of ISGs proteins were potently upregulated in this model, dozens of proteins like MED1, PUM2, MUC1, VPS26B, MRC1, CD59, NPLOC4, S100A14, and S100A8 were significantly downregulated. These data indicated that strong but controllable innate immune response was elicited for SARS-CoV-2 defense and clearance in the COVID-19 model, which provide evidence to explain why CMs mainly present mild symptom after SARS-CoV-2 infection.

### No Thrombopenia Was Observed in SARS-CoV-2–Infected CMs

PLTs, as dynamic cells, participate in inflammation and prothrombotic responses in many viral infections (49). Thrombopenia, caused by reduced PLTs production and unrestricted consumption, has been reported to be associated with enhanced risk of severe COVID-19 and mortality (50). Our data demonstrated that no thrombopenia was observed in moderate CMs models.

Platelet factor 4 (PF4) and platelet-expressing chemokines pro-platelet basic protein (PPBP) were reported significantly downregulated in severe COVID-19 patients, which associated with platelet degranulation and potently contributed to thrombopenia (5). Herein, neither PF4 (1.27-fold) nor PPBP (1.78-fold) were decreased in lung tissues of infected CMs. Whereas 17 proteins which associated with platelet aggregation and function were altered significantly. Of them, KNG1, TXNIP, ITIH4, TIMP1 and SERPING1/2/3, which contribute to platelet aggregation and function (51), were increased; whereas ITGA1, CLIC5, ARRB1, MFAP2, and GNAI1 (52–54), which negatively regulate platelet activation, were greatly decreased (**Figure 6A**). As to these proteins, TIMP1 was reported to be overexpressed in anti-inflammatory M2 macrophages upon SARS-CoV-2 infection (55). TXNIP regulates neutrophil platelet aggregates in acute lung injury (ALI), and has been proved to be involved in glucose and lipid metabolism, and NLRP3 inflammasome activation, a known factor for the immunopathogenesis of COVID-19 (56). CLIC5 could be exploited by virus for transportation of new virions (57).

### Metabolomic Changes in the Sera of SARS-CoV-2–Infected CMs

Compared with the normal control, more than 116 metabolites, or their derivatives, were significantly decreased in the sera of SARS-CoV-2 infected-CMs. Enriched are 11 metabolites mainly involved in lipids, bile acids, amino acids, and heterocyclic compounds (**Figure 7A**).

According to the aforementioned data, we analyzed the metabolites closely associated with platelet degranulation, innate immune response, neutrophil and macrophage function. We found that several well-known metabolites, such as serotonin, choline, mannose, arachidonate, and eicosanoids (prostaglandins, thromboxanes, and leukotrienes) (58–60), were not changed significantly in infected-CMs. Consistent with previous reports in COVID-19 patients (61, 62), dozens of metabolites like L-cystine, L-glutamic acid, and L-serine were increased in COVID-19 CMs as compared with the healthy control. Notably, for the first time, several lipids, nucleotide, bile acids, and amino acids metabolites were found to be apparently changed, including different products of carnitine, glutathione (0.3-fold), N-acetyl-L-glutamic acid (2.1-fold), adenosine (0.02-fold), and inosine (2.99-fold) (**Figure 7B**).

It is well known that adenosine play critical roles in regulating neutrophil chemotaxis, phagocytosis, and granule release (63, 64). Usually, pathological conditions of inflammation lead to release of adenosine, conferring protection against tissue damage, platelet activation and inflammatory response. In addition, adenosine could be phosphorylated to AMP or deaminated to inosine (65). We found that adenosine decreased to 0.02-fold and inosine increased up to 2.99-fold in SARS-CoV-2 infected CMs. This revealed that adenosine generated and released by neutrophils at sites of inflammation was mainly deaminated to inosine, which was consistent with the increased platelet activation, recruitment and activation of neutrophils and leukocytes observed in the COVID-19 model in CMs. In addition, decreased adenosine-to-inosine ratio indicated elevated adenosine deaminases (ADARs) activity. ADARs can be a pro- or anti-viral factor and has been reported to shape SARS-CoV-2 fate by virus genome editing (66). Thus, these data suggested that elevated ADAR (1.39-fold) are involved in the moderate COVID-19 model.

Carnitine derivatives are the most changed metabolites, among which 38 metabolites were significantly decreased, with lowest down to 0.28-fold. Besides the role in β-oxidation, carnitine also acts in immune regulation as an anti-inflammatory and anti-apoptotic factor (67). It has been reported that carnitine negatively regulated neutrophil and platelet activation (68), and decreased serum carnitine levels usually associated with impaired reactions, metabolic disorders and recurrent infections (69). Thus, significant carnitine reduction in CMs might relieve immune suppression and profit SARS-CoV-2 clearance.

To further uncover the function of DEGs and metabolites in this model, a protein-metabolite interaction regulatory network was done. As shown in **Figure 7C**, most metabolites are associated with metabolic pathways, bile acid biosynthesis and secretion pathways, and lipid metabolism in SARS-CoV-2 infected CMs, and there are extensive and fine regulations between metabolites and differentially expressed proteins. The detailed mechanism and function of these intermodulations is still under investigation.

## Discussion

COVID-19, an unprecedented threat caused by SARS-CoV-2, has been spreading worldwide rapidly. Until now, over 523 million individuals have been infected, leading to over 6.279 million deaths. Epidemiological and clinical characteristics studies have uncovered that most of COVID-19 patients usually recovered with good prognosis who displayed mild or moderate symptoms, or even a higher proportion of asymptomatic symptoms in Delta or Omicron infections (70, 71). However, it is still a great challenge to discriminate cases which will likely become clinically severe and to treat them effectively. Although many specific hematologic indicators have been reported to be strongly associated with severe COVID-19 cases, with low levels of PLTs count, lymphocyte count and percentage, total protein, and high levels of D-dimer, leukocyte count, CRP, creatinine, neutrophil count and percentage, creatine kinase activity, and prolonged prothrombin time to be changed the most (30, 72, 73), the pathogenesis and detailed mechanisms of COVID-19, especially at early stage of infection is still largely unknown.

In the present study, we analyzed the lung tissues and sera samples of SARS-CoV-2–infected CMs *via* 4D Label-free proteomics strategy and UPLC-MS/MS targeted metabolomics approaches. About 4493 proteins and 500 metabolites were identified and quantified. KEGG analysis suggested that most of these differentially expressed proteins focused on innate immune response, neutrophil recruitment, activation and degranulation, and platelet degranulation.

Innate immune response stands as the first line of antiviral defense, the cellular innate immune responses play crucial roles in initiating resistance to virus infection. SARS-CoV-1–infected patients elicited strong innate inflammatory responses, rather than a virus-specific immune response (74). Although only a small percentage of primary endothelial cell were infected, Dengue Virus exerts substantial influence on type I IFN-driven innate immune response induction that can effectively restrict infection (75). Therefore, innate immunity may be the best weapon for flattening the curve of COVID-19 spread, and dysregulated immune response can reduce the ability to detect antigens, blunt the healing process and delay the recovery of patients with COVID-19 (76). In our model, dozens of known host genes that associated with innate immunity and response to viral infections, such as MX1/2, ISG15, IFIT1, DDX58/60, IFI44L, IFI44, OAS3, STAT1, POLB, BANF1, TRIM28, LOC703156, MED1, PUM2, VPS26B, MRC1, CD59, NPLOC4 and S100A14/A8, were greatly changed. Several of these genes can regulate viral genome replication, viral infection process and life cycle. The significant change of ISG15, MX1, MX2, and STAT1 strongly indicated that IFN signaling pathway associated anti-viral action play critical roles in SARS-CoV-2–infected CMs model (77, 78). Which is consistent with a previous report that IFN signaling is significantly induced in rhesus macaque infected lungs (79).

In this moderate COVID-19 model, we found that neutrophils play pivotal roles in host defense against SARS-CoV-2 infection. The proteomics and Luminex data showed that various macrophage and neutrophil related cytokines and chemokines like MCP-1, IP-10 (CXCL10), MIG, MIP-1β (CCL4), CXCL8 (IL-8), SAA2, and AXL were dramatically upregulated at 7 days after infection. This indicated that the circulating or tissue resident macrophages are activated by the intruder/virus at the fastest time and recruited neutrophils to the attack site where they synergistically defense virus infection. IP-10, a chemoattractant for monocytes/macrophages, has been reported as an excellent predictor for the progression of COVID-19 strongly associated with disease severity and ICU admission (80). Our data supported that IP-10 also contributed to the progression of COVID-19 in infected CMs. Most nucleated cells, including neutrophils and monocytes, are the main source of IL-8. So, it is easy to understand the high level of IL-8 after SARS-CoV-2 infection. According to our data, we hypothesize that SARS-CoV-2 infection activates innate immune cells to secrete multi-factors at early stage in CMs, which then exert anti-viral effects. Among them, the sedentary macrophages in lung tissues released abundant IL-8, which acts as a classical neutrophil chemotactic factor, recruiting neutrophil from the blood stream into the infected tissue (alveolar space and airways). IL-8 stimulated the phagocytosis and degranulation of neutrophil to kill virus, and induced the necrosis and exhaustion of neutrophil as well. Neutrophilia is a main feature of severe COVID-19 cases, whereas neutropenia is found in the sera of our model (apparent neutrophil infiltration were also observed in lung tissues). This may be partly due to the significantly regulated metabolites like adenosine, inosine and carnitine derivatives, which take part in regulating neutrophil chemotaxis, phagocytosis, and granule release.

A recent study reported that immature neutrophils with elevated calprotectin S100A8/S100A9 plasma levels can be used as robust biomarkers of COVID-19 severity (81). In the current model, S100A8 was significantly downregulated, and no apparent change was found for S100A9. In addition, the classical inflammatory cytokines secreted by neutrophils and macrophages like TNF-α, IL-1β and IL-2 were not obviously changed, whereas the immunosuppressive IL-10 was significantly upregulated. Which would be helpful to explain the moderate “cytokines storm” and tissue injury in the model and we speculated that potent but controllable spatial and temporal innate immune cell response was essential for SARS-CoV-2 defense and clearance (82), and might be used as potential biomarkers for discriminating severe from mild or moderate COVID-19.

Our Luminex assay data showed that CD40L was significantly increased in CMs, which was also found greatly elevated in ICU patients (83). Although CD40L is a marker of platelet and T-cell activation, the major source of CD40L present in plasma is derived from platelet, thereby the increase of CD40L here suggested that platelet activation might be an important factor in the pathophysiology of COVID-19–associated coagulopathy and might accelerate the progression of disease. Consistently, proteomic and metabolomics data from CMs revealed several genes (KNG1, TXNIP, ITIH4, TIMP1, SERPING1/2/3, ITGA1, ADAR, and CLIC5 etc.) and molecules (adenosine and inosine) were related with platelet activation, thus, adding antiplatelet might be a possible therapeutic for COVID-19.

As to other viral immunity responses, complement activation can suppress virus invasion and trigger inflammation. Suppression of complement system has successfully ameliorated syndrome of SARS-infected mouse model (84). Upregulation of C1s (1.60-fold), C1q (1.53-fold), C2 (2.76-fold), C3 (1.9-fold), and C4b (2.45-fold) indicated that timely complement activation may contribute to COVID-19 in CMs.

Since some mild patients clear SARS-CoV-2 virus without obvious symptoms stands in sharp contrast to the lethal damage that the virus has brought upon severe patients, early diagnosis and treatment of severe COVID-19 patients remain a major challenge. Therefore, it is necessary to elucidate the pathogenesis and detailed mechanism of mild COVID-19 casels, especially at early stage of SARS-CoV-2 infection. Our data demonstrated that innate immune system, neutrophil and platelet activation and degranulation play central roles in these processes in CMs, which mimicked moderate COVID-19 syndrome. This study shed light on characteristic protein and metabolite changes in COVID-19 and might be used as potential biomarkers reservoir for severity evaluation.

## Data Availability Statement

The data presented in the study are deposited in the ProteomeXchange repository, accession number PXD034589.

## Ethics Statement

The animal study was reviewed and approved by the Administrative Committee on Animal Welfare of the Institute of Military Veterinary.

## Author Contributions

Designed and supervised the project: HW, XX, YL, and YG. Conducted the experiments: TW, FM, LL, LH, JZ, CZ, GY, KM, ZW, BW, RS, WL, NL, and DL. Collected the original data and conduct statistical analysis: SL, FW, ZZ, GF and HX. Conducted proteomic and metabolomics analysis: YL, XX, and YG. Wrote the manuscript with input from co-authors: XX, YL, FW, and YG.

## Funding

This work was supported by grant from the National Key Research and Development Project of China (No.2021YFC2301701), the National Natural Science Foundation of China (No. 31972720) and the Ministry of Science and Technology of China (No. 2020YFC0846100).

## Conflict of Interest

The handling Editor LR declared a shared (parent) affiliation with the authors SL, FW, RS, WL, JZ.

The remaining authors declare that the research was conducted in the absence of any commercial or financial relationships that could be construed as a potential conflict of interest.

## Publisher’s Note

All claims expressed in this article are solely those of the authors and do not necessarily represent those of their affiliated organizations, or those of the publisher, the editors and the reviewers. Any product that may be evaluated in this article, or claim that may be made by its manufacturer, is not guaranteed or endorsed by the publisher.
